# Bisphenol A Exposure Enhances Atherosclerosis in WHHL Rabbits

**DOI:** 10.1371/journal.pone.0110977

**Published:** 2014-10-21

**Authors:** Chao Fang, Bo Ning, Ahmed Bilal Waqar, Manabu Niimi, Shen Li, Kaneo Satoh, Masashi Shiomi, Ting Ye, Sijun Dong, Jianglin Fan

**Affiliations:** 1 Key Laboratory of Urban Environment and Health, Institute of Urban Environment, Chinese Academy of Sciences, Xiamen, People’s Republic of China; 2 Department of Molecular Pathology, Interdisciplinary Graduate School of Medicine and Engineering, University of Yamanashi, Yamanashi, Japan; 3 Department of Laboratory Medicine, Interdisciplinary Graduate School of Medicine and Engineering, University of Yamanashi, Yamanashi, Japan; 4 Institute for Experimental Animals, Kobe University Graduate School of Medicine, Kobe, Japan; University of Missouri, United States of America

## Abstract

Bisphenol A (BPA) is an environmental endocrine disrupter. Excess exposure to BPA may increase susceptibility to many metabolic disorders, but it is unclear whether BPA exposure has any adverse effects on the development of atherosclerosis. To determine whether there are such effects, we investigated the response of Watanabe heritable hyperlipidemic (WHHL) rabbits to 400-µg/kg BPA per day, administered orally by gavage, over the course of 12 weeks and compared aortic and coronary atherosclerosis in these rabbits to the vehicle group using histological and morphometric methods. In addition, serum BPA, cytokines levels and plasma lipids as well as pathologic changes in liver, adipose and heart were analyzed. Moreover, we treated human umbilical cord vein endothelial cells (HUVECs) and rabbit aortic smooth muscle cells (SMCs) with different doses of BPA to investigate the underlying molecular mechanisms involved in BPA action(s). BPA treatment did not change the plasma lipids and body weights of the WHHL rabbits; however, the gross atherosclerotic lesion area in the aortic arch was increased by 57% compared to the vehicle group. Histological and immunohistochemical analyses revealed marked increases in advanced lesions (37%) accompanied by smooth muscle cells (60%) but no significant changes in the numbers of macrophages. With regard to coronary atherosclerosis, incidents of coronary stenosis increased by 11% and smooth muscle cells increased by 73% compared to the vehicle group. Furthermore, BPA-treated WHHL rabbits showed increased adipose accumulation and hepatic and myocardial injuries accompanied by up-regulation of endoplasmic reticulum (ER) stress and inflammatory and lipid metabolism markers in livers. Treatment with BPA also induced the expression of ER stress and inflammation related genes in cultured HUVECs. These results demonstrate for the first time that BPA exposure may increase susceptibility to atherosclerosis in WHHL rabbits.

## Introduction

Bisphenol A (BPA) is one of the world's most produced chemicals and is widely used as a key monomer for the production of polycarbonate plastic and epoxy resins [1]. These two commercial materials have been commonly used in the manufacture of various consumer goods, industrial products, and medical devices [2], [3]. Given the prevalence of BPA in our environment and daily lives, it can be detected in serum, urine, breast milk and saliva in the majority of populations in different countries [1], [2], [4]. Thus, concern has grown regarding whether BPA exposure can cause health problems in humans [3], as underscored by recent cross-sectional and longitudinal studies that show that urinary or serum levels of BPA are positively associated with various cardiovascular diseases (CVD). An epidemiological study analyzed data from the 2003–2004 National Health and Nutrition Examination Survey (NHANES) and was the first to report positive associations between higher urinary BPA concentrations and increased risks of cardiovascular diseases, including angina, coronary heart disease and heart attacks [5]. Other studies also found significant positive associations, which were independent of traditional CVD risk factors, between urinary BPA levels and coronary heart diseases using data from the 2003–2006 NHANES database and the European Prospective Investigation of Cancer in Norfolk, UK, in the 1990s [6], [7]. Recent epidemiological studies have also shown that either urinary or serum BPA levels were positively associated with coronary artery stenosis [8], carotid atherosclerosis [9], and peripheral arterial disease [10], suggesting that BPA exposure may be an emerging risk factor for the development of atherosclerosis. However, this latter hypothesis has not been verified experimentally using appropriate animal models. This is an important issue because it is not clear whether BPA exposure is causal for the development of atherosclerosis. In fact, the toxicological mechanisms of BPA in terms of atherosclerosis remain largely unknown [11]. Several studies have shown that BPA exposure increases atherosclerosis in mice [12], [13] and alters cardiac functions in both mice and rats [14]–[16].

Although these rodent studies are informative, it is not known whether these results can be extrapolated to humans because rodents are quite different from humans in terms of lipid metabolism, glucose metabolism, cardiovascular systems, and responses to inflammatory stimuli [17]–[19]. Furthermore, regional distributions of fat depots, their cellular compositions (e.g., brown vs. white fat, infiltration by macrophages), and regulations of resistin, agouti protein, adipsin, and adrenergic receptors are dissimilar between rodents and humans [20]. In this regard, there are advantages to studying lipid metabolism and atherosclerosis in rabbits rather than mice [17]. In addition, rabbits are phylogenetically closer to humans than are rodents [17]. Like humans but unlike rodents, rabbits are LDL-mammals and have plasma-cholesteryl-ester-transfer proteins, which are important regulators for lipid metabolism [17], [18]. Furthermore, the phenotypes and pathogenesis of atherosclerotic lesions in rabbits are similar to those observed in human atherosclerosis [17]. WHHL rabbits are genetically deficient in low-density lipoprotein receptors due to spontaneous 4-amino-acid deletions in the cysteine-rich ligand-binding domains in exon-4 positions of LDL receptors [21], [22]. Even when fed chow diets, which have been extensively used for the study of hypercholesterolemia, atherosclerosis, and insulin resistance, WHHL rabbits are characterized by hyperlipidemia and spontaneous atherosclerosis in both aorta and coronary arteries [23]–[25].

To examine whether BPA exposure plays any role in the development of atherosclerosis, we administered BPA daily at a dose of 400 µg/kg body weight (BW) to WHHL rabbits for 12 weeks. We found that BPA treatment enhanced atherosclerosis both in the aortic arch and coronary arteries. To the best of our knowledge, this is the first report using non-rodent models to investigate BPA exposure and atherosclerosis in the setting of hyperlipidemia.

## Materials and Methods

### Animals

Fourteen-week-old, post pubertal male WHHL rabbits provided by Kobe University (Kobe, Japan) were randomly divided into 2 groups (designated as vehicle and BPA-exposure groups, n = 6 for each group) and raised in a facility with constant temperature (25±2°C) and a 12∶12-hr light:dark cycle controlled strictly by an automatic device. Stainless-steel cages, automatic waterers and food containers were used in this study. All animals could access water *ad libitum* and were fed 100 g of a restricted standard chow diet (CR-3, CLEA Japan, Inc., Tokyo) every day. Daily food intake and weekly body weight were monitored strictly.

BPA (97% pure) was purchased from Sigma-Aldrich (St. Louis, MO, USA) and was first dissolved in ethanol and then diluted in corn oil (Wako Chemical Industries Co., Osaka, Japan). BPA solution (400 µg/kg for the exposure group) was administered by gavage every morning for 12 weeks. The vehicle group was administered the same volume of corn oil without BPA. The polypropylene tubes which are regarded as BPA free [26] were used for all procedures. A dose of 400 µg/kg was chosen for the following two reasons: (1) a previous study as well as preliminary experiments in our laboratory showed that monkeys, mice and Japanese white rabbits exposed to this dose exhibited average unconjugated 24-hr serum BPA levels that could be within the range observed in the general human population [27] and (2) based on the similarity of BPA pharmacokinetics between humans, monkeys and mice, we estimated the maximal daily human exposure to BPA via multiple routes to be approximately 500 µg/kg/day [27]–[29].

Before the experiment, we investigated serum BPA kinetics in WHHL rabbits within 24 hours of oral BPA administration. For this purpose, blood was collected at 0 (pre-feeding), 0.5, 1, 2, 4, and 24 hours after the rabbits were administered 400 µg/kg BPA by gavage. For BPA residual accumulation analysis, blood samples were collected biweekly from middle-ear arteries before gavage administration. All animal experiments were performed with the approval of the Animal Care Committee of the University of Yamanashi and complied with the Guide for the Care and Use of Laboratory Animals published by the US National Institutes of Health [30].

### Measurements of serum BPA levels

Blood samples were allowed to stand at room temperature for 15 min to enable clotting, and then serum was obtained after centrifugation at 3,000 rpm for 20 min at 4°C. Serum was kept at −80°C until use. Serum BPA levels were measured by detecting unconjugated BPA using a Shimadzu Prominence LC-20A series high-performance liquid chromatography (HPLC) (Shimadzu, Kyoto, Japan) in conjunction with tandem mass spectrometry (MS/MS) (Applied Biosystems, Foster City, CA, USA) according to a previously described method [31]. The mean extraction recovery of BPA through the entire analytical procedure ranged from 81% to 113%. The BPA detection limits of this method were 0.02∼0.27 ng/mL. The details of this analytical procedure are shown in Technical Description S1. Serum BPA pharmacokinetic parameters, including the maximum attained value (C_max_), the terminal-phase half-life time (t_½_), and the average of the area under the curve (AUC) for 24 hr after gavage (Average AUC_0–24_) were calculated according to methods described elsewhere [27].

### Analysis of blood biochemical parameters

Blood was collected from rabbits after 16 hr of fasting. The plasma levels of total cholesterol (TC), HDL-cholesterol (HDL-C), triglycerides (TG), and LDL-cholesterol (LDL-C) were measured using Wako assay kits (Wako Pure Chemical Industries, Osaka, Japan) according to established methods described in previous studies [30]. The plasma glucose concentrations were also measured using Wako glucose assay kits following the manufacturer’s instructions.

### Analysis of serum cytokines levels

Blood was allowed to stand at room temperature for 15 min to clot and then serum was obtained after centrifugation at 3,000 rpm for 20 min at 4°C. The serum cytokines levels of TNF-α and IL-6 were analyzed using rabbit TNF-α and IL-6 ELISA kits (Cloud-Clone Corp. and USCN, Houston, USA) according to the manufacturer’s instructions. The details of procedures are shown in Technical Description S1.

### Analysis of aortic atherosclerosis

At the end of the experiments, all rabbits were euthanized by injection of an overdose of sodium pentobarbital solution (100 mg/kg). Incidents of aortic atherosclerosis were quantified according to a previously described method [32]. The aortic trees were isolated and fixed in 10% buffered formalin. For analysis of the gross sizes of atherosclerotic lesions, whole aortas were stained with Sudan IV and photographed using a digital camera, after which sudanophilic areas were measured using an image analysis system (WinROOF ver. 7.0, Mitani Corporation, Tokyo, Japan). For histological analysis, the aortic arch was cut into 10 sections (3-µm-thick) and stained with hematoxylin and eosin (H&E) and Elastica van Gieson (EVG). To visualize the cellular components in the lesions, serial paraffin sections of each arch were immunohistochemically stained with monoclonal mouse antibodies (mAbs) against either macrophages (RAM11, Dako, working dilution 400×) or α-smooth muscle actin (HHF35, Dako, working dilution 200×) for smooth muscle cells (SMCs) [30], [32]. Positively immunostained areas of macrophages (Mφ) and SMCs in the lesions were quantified using an image analysis system and were expressed as percentages.

### Analysis of coronary atherosclerosis

To assess coronary atherosclerosis, the left coronary artery roots were cut into 4 slices (3-µm-thick) in 50-µm intervals, and a total of 40 slices were sectioned serially. To analyze the lesions of coronary arteries, 10 serial sections were stained with H&E, and the lesions were observed under a light microscope, quantified using an image analysis system and expressed as the stenosis (%) of the lumen area [lesion area/(total lumen area)×100%] based on the method described previously [32]. To visualize the cellular components in the lesions, each of 10 serial sections was immunohistochemically stained with mAbs against Mφ and SMCs, as described above.

### Pathological and morphometric analysis

Liver, heart, and adipose tissue (including subcutaneous and visceral regions) were collected and weighed when wet. In each case, the heart and the small pieces of liver and adipose tissue were fixed in 10% buffered formalin. The heart was sectioned into 5 blocks, and 3 sections were cut from each block at 500-µm intervals and then stained with H&E for analysis. For liver and adipose tissues, 3-µm-thick sections were cut serially and stained with H&E for histological analyses. Morphometric analysis of adipose tissue was conducted using a method described previously [30]. In brief, more than 300 adipocytes of each section were selected randomly, and cellular diameters were measured using an image analysis system (WinROOF ver. 7.0). Each block of heart was observed under a light microscope using H&E-stained sections, and the lesion areas in the cardiac muscle were quantified via the image analysis system.

### Real-time RT-PCR analysis

A piece of liver was quickly frozen in liquid nitrogen, and total RNA was isolated using Trizol reagent (Invitrogen, Carlsbad, CA, USA). Hepatic expression of genes related to ER stress, lipid metabolism, and inflammation [ER stress, including C/EBP homologous protein (Chop), binding immunoglobulin protein (Bip), and calreticulin (CALR); lipid metabolism, including peroxisome proliferator-activated receptor alpha (PPAR-α), liver X receptor alpha (LXR-α), and microsomal triglyceride transfer protein (MTTP); inflammation, including interleukin-1 beta (IL-1β), plasminogen activator inhibitor-1(PAI-1), and interleukin-6 (IL-6)] were analyzed using real-time quantitative reverse transcription polymerase chain reaction (qRT-PCR) using SYBR Premix Ex TaqTM kits (Takara, Tokyo, Japan) following the manufacturer’s instructions. Rabbit glyceraldehyde 3-phosphate dehydrogenase (GAPDH) was used as an internal standard for relative quantification, and the relative amount of mRNA was calculated using the 2^−ΔΔCT^ method [33]. The primers used in this experiment are shown in Table S1.

### Cell-culture experiments

To investigate the possible molecular mechanisms induced by BPA, we also performed cultured cell studies using human umbilical-cord-vein endothelial cells (HUVECs) (Cell Bank, Chinese Academy of Sciences, Shanghai, China) and rabbit aortic smooth muscle cells (SMCs). The details of the cell experiments are contained in Technical Description S1. Total RNA was extracted and qRT-PCR was performed as reported previously [33]. We examined the effects of BPA treatment on the gene expression of Bip, monocyte chemotactic protein-1 (MCP-1), nuclear factor-kappa B (NF-κB), tumor necrosis factor alpha (TNF-α), vascular cell-adhesion molecule-1 (VCAM-1), and vascular endothelial growth factor A (VEGF-A) on the HUVECs at 24 hr. The procedure and the primer sequences are shown in Technical Description S1 and Table S1. Meanwhile, the proliferation of SMCs caused by BPA treatment for 24 hr was also assessed using cell counting kit-8 (Dojindo Molecular Technologies, Inc., Rockville, USA) following the manufacturer’s instructions. The details of procedures are also shown in Technical Description S1.

### Statistical Analysis

Statistical analysis was performed using SPSS 16.0 for Windows software (SPSS Inc., Chicago, IL, USA). The Mann-Whitney U test was used for nonparametric analysis of the positive areas of SMCs and macrophages (Mφ) in the aortic lesions. The Student’s t-test was used to compare the results of other assays. In each case, statistical significance was indicated by *p*<0.05.

## Results

### Serum levels of BPA

**Figure 1:**
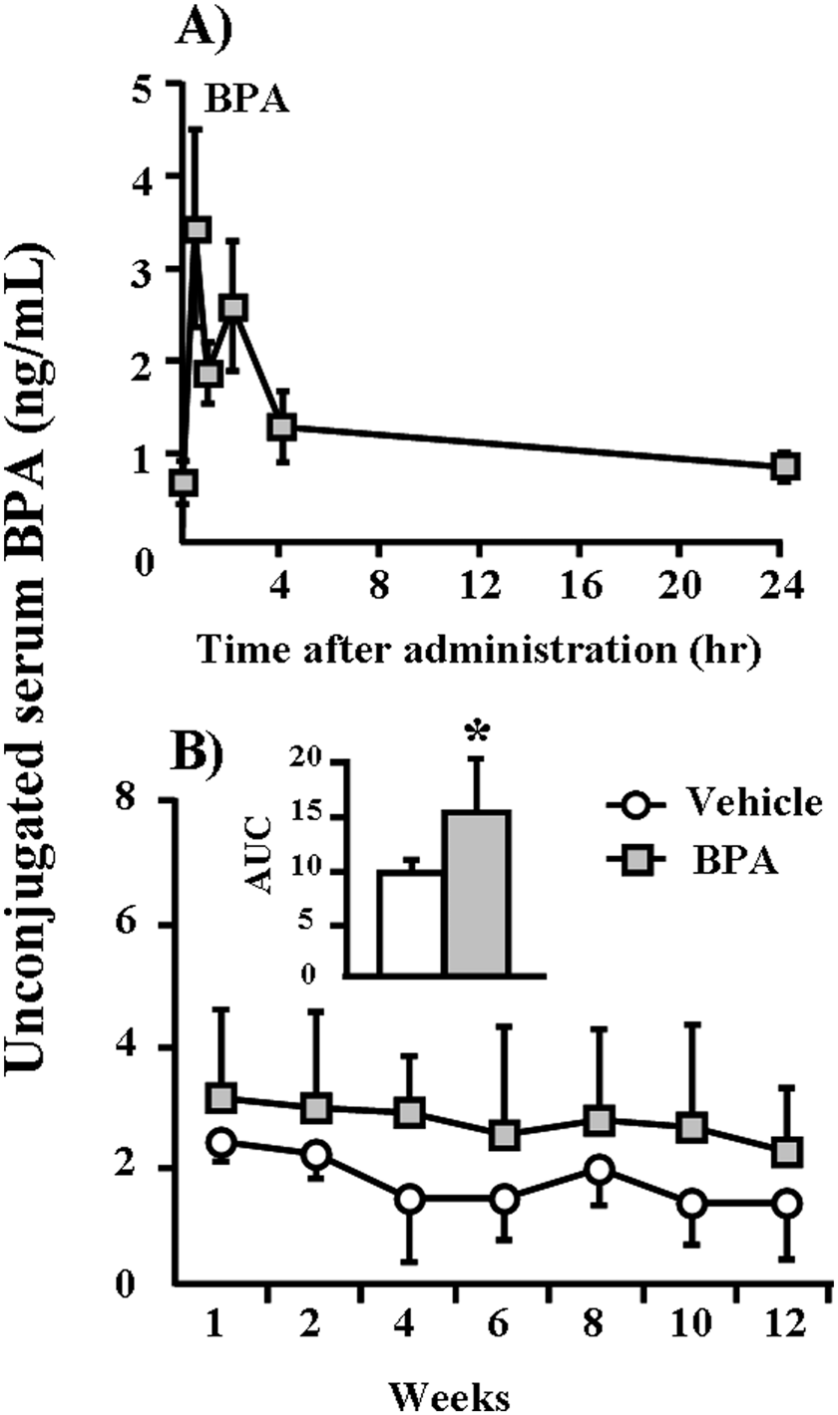
Kinetic changes and accumulation of serum BPA concentrations in WHHL rabbits. (A) WHHL rabbits were fed BPA (400 µg/kg) by gavage, and serum levels of BPA were measured over the next 24 hr as described in the Materials and Methods section. Serum levels of BPA are expressed as the means ± SE. For each group, n = 6. (B) Serum levels of BPA were measured biweekly over the course of 12 weeks as described in the Materials and Methods section. Serum levels of BPA are expressed as the means ± SD, n = 6 for each group. Total exposure volumes during experiments are calculated as the areas under the curves (AUC’s) (shown in the insert). *p<0.05 vs. vehicle.

Because no data were available in the published literature, we first investigated the kinetics of serum BPA in WHHL rabbits after one session of BPA feeding. After gavage feeding of 400 µg/kg BPA, serum levels of BPA peaked within 1 hr and declined to normal levels at 24 hr (Figure 1A), suggesting that BPA can be rapidly absorbed and catabolized in rabbits. We also compared the serum BPA kinetics of WHHL rabbits with those of other species reported in the literature after BPA (400 µg/kg) exposure [27], [34]. The C_max_ of unconjugated BPA in the serum of WHHL rabbits reached 3.41 ng/mL at 0.5 hr and declined sharply close to the initial level of 0.83 ng/mL at 24 hr (Figure 1A). The average AUC value of unconjugated BPA from 0 to 24 hr (1.22 ng/mL) in WHHL rabbits was slightly higher than values obtained in adult monkeys and mice (Table 1).

**Table 1:** Serum pharmacokinetic parameters for unconjugated BPA in WHHL rabbits compared with monkey and mice.

Species	Gender	Age	BPA (µg/kg)	C_max_ (ng/mL)^a^	Terminalt½ (hr)^b^	AverageAUC_0–24_ (ng/mL)^c^	References
WHHL rabbit	Male	Adult	400	3.41	18.44	1.22	This study
CD-1mice	Female	Adult	400	3.28	33.64	0.70	[27]
Rhesusmonkey	Female	Adult	400	3.95	8.88	0.52	[27]
CD-1mice	Female	Neonatal	395	14.82	9.35	2.78	[34]

a C_max_: maximum attained value; ^b^Terminal t½: the terminal phase half-life;

c AUC_0–24_: the area under the curve at *t* = 24 hr after gavage.

**Figure 2:**
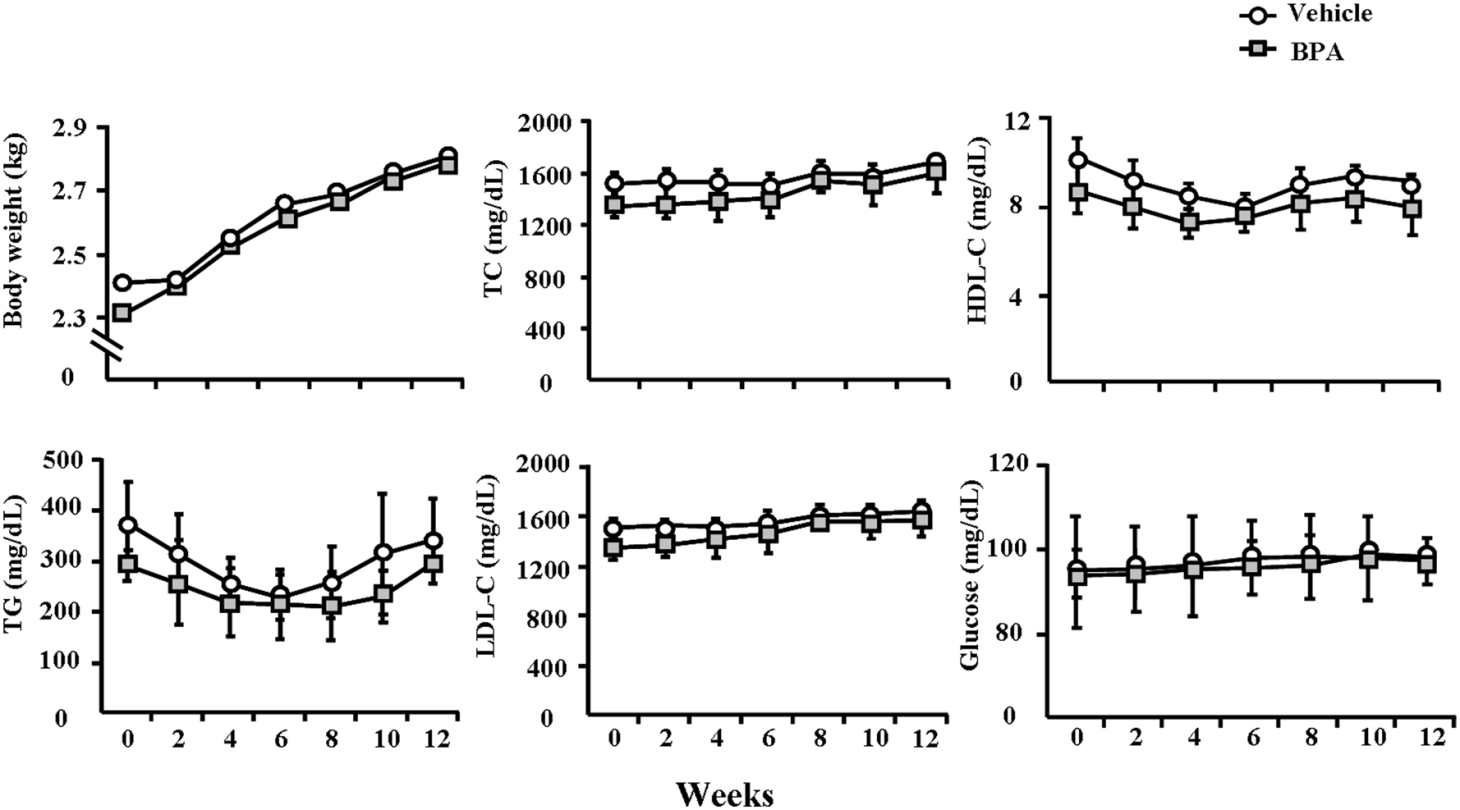
Body weights, serum lipids and glucose levels in WHHL rabbits over 12 weeks. These indexes were monitored every 2 weeks. Data are expressed as the means ± SD, n = 6 for each group.

On the basis of serum BPA kinetics in WHHL rabbits shown above, we subsequently measured BPA serum levels at different weeks and examined whether any residual BPA accumulated in the serum when BPA was given daily for 12 weeks. As shown in Figure 1B, the serum BPA levels of vehicle WHHL rabbits were approximately 1.3∼2.3 ng/ml (similar to those of normal humans (0.5∼2.0 ng/mL) [1]. However, BPA administration led to a slight increase in serum BPA in WHHL rabbits (2.2∼3.1 ng/mL) during 12 weeks. Although there were no statistically significant differences at each time point between BPA-treated and vehicle groups, the total amounts of BPA expressed by AUC were significantly higher (*p* = 0.02) in the BPA-exposure group compared to the vehicle group. BPA exposure did not significantly affect body weight and plasma lipid and glucose levels in the BPA-exposure group compared to the vehicle group (Figure 2).

### Serum cytokines levels

**Figure 3:**
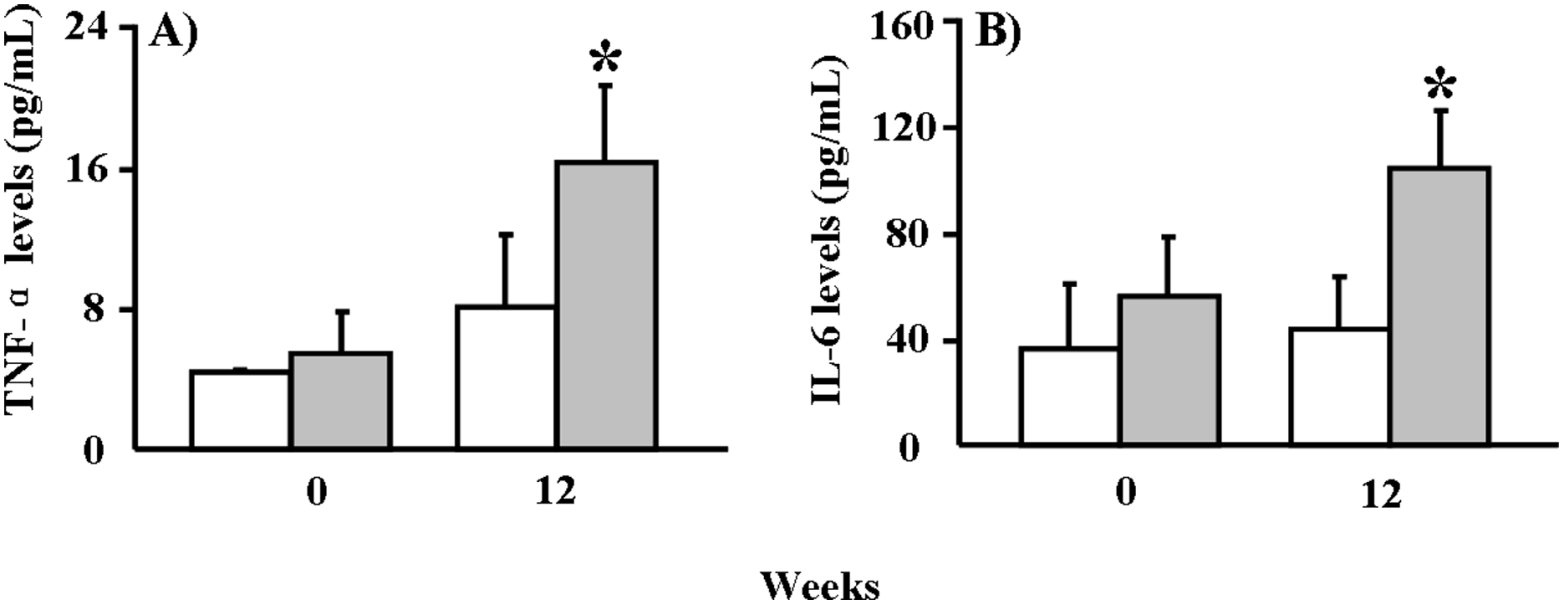
ELISA analysis of serum cytokines levels in WHHL rabbits. (A) TNF-α serum concentrations at 0 and 12 weeks. (B) IL-6 serum concentrations at 0 and 12 weeks. Data are expressed as the means ± SD, n = 6 for each group. *p<0.05 vs. vehicle.

The serum levels of TNF-α and IL-6 were increased by 1.03-fold and 1.37-fold in the BPA group compared to the vehicle group at 12 weeks (Figure 3).

### Aortic and coronary atherosclerosis

**Figure 4:**
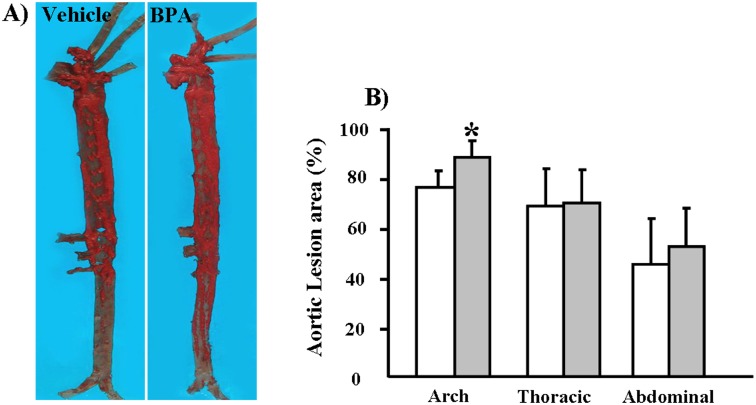
Quantitative analysis of aortic atherosclerosis in WHHL rabbits at 12 weeks. (A) Representative micrographs of the aortic trees stained with Sudan IV. (B) The sudanophilic en-face lesion areas in different parts of aortic trees were determined using an image analysis system as described in the Materials and Methods section. Data are expressed as the means ± SD, n = 6 for each group. *p<0.05 vs. vehicle.

**Figure 5:**
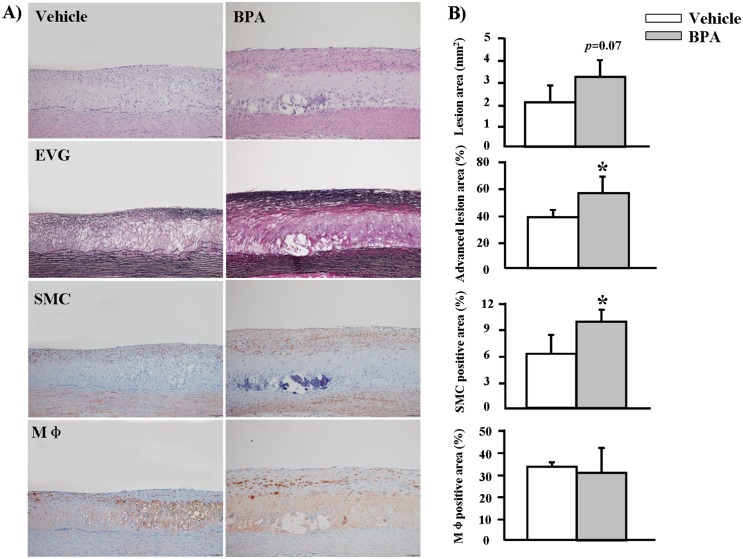
Microscopic analysis of atherosclerosis in the aortic arches of WHHL rabbits at 12 weeks. (A) Representative micrographs stained with H&E and EVG for intimal lesion analysis are shown in the top two panels. Representative micrographs stained with HHF35 and RAM11 mAbs for immunohistochemical analysis of SMCs and macrophages are shown in the lower two panels. (B) Intimal lesions, advanced lesions, SMCs and Mφ-positive areas of the sections were quantified using an image analysis system as described in the Materials and Methods section. Data are expressed as the means ± SD, n = 6 for each group. *p<0.05 vs. vehicle. Original magnification: 10×.

**Figure 6:**
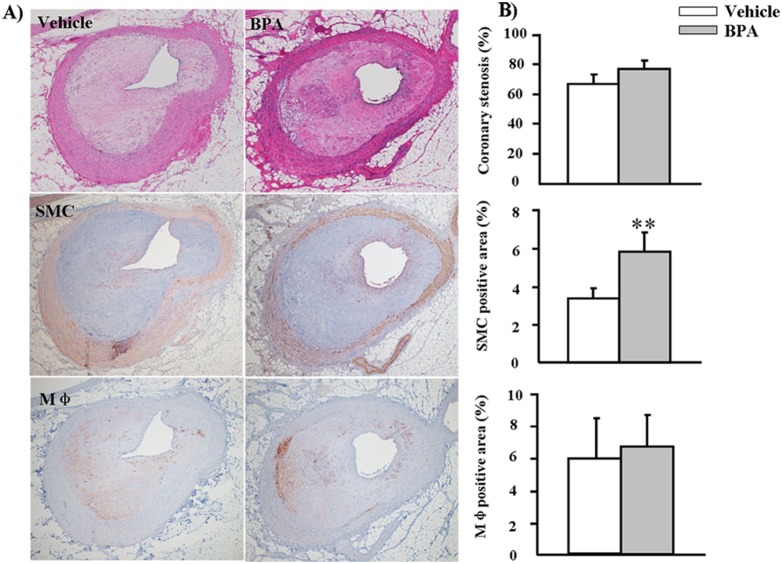
Microscopic analysis of atherosclerosis in the left coronary arteries of WHHL rabbits at 12 weeks. (A) Representative micrographs stained with H&E for coronary stenosis analysis are shown in the top panel. Representative micrographs stained with HHF35 and RAM11 mAbs for immunohistochemical analysis of SMCs and Mφ are shown in the middle and lower panels. (B) Coronary stenosis, SMCs and Mφ-positive areas of the sections were quantified using an image analysis system as described in the Materials and Methods section. Data are expressed as the means ± SD, n = 6 for each group. **p<0.01 vs. vehicle. Original magnification: 4×.

We found that, compared to the vehicle group, the gross lesion area in the BPA group was significantly increased by 17% (*p* = 0.04) in the aortic arch and that thoracic and abdominal aortic lesions were increased by 1.7% and 15%, respectively, but the latter effects were not statistically significant (*p*>0.05) (Figure 4). We quantified the microscopic lesion areas of the aortic arch and found a 57% increase in the lesions of WHHL rabbits (*p* = 0.07) compared to the vehicle group (Figure 5). Immunohistochemical staining showed that the increased lesion areas in the BPA-exposure group were characterized by marked increases in smooth muscle cells (60%, *p* = 0.03), whereas Mφ numbers did not differ significantly between the two groups (Figure 5). Furthermore, advanced lesion areas (advanced lesions, referring to those lesions that contain a fibrotic cap and a lipid or necrotic core with or without calcification) [35] were increased by 37% (*p* = 0.04) compared to the vehicle group. In addition to effects in the aorta, evidence of coronary stenosis increased by 11% (*p*>0.05) in the BPA-exposure groups compared to the vehicle group. Likewise, smooth muscle cells increased by 73% (*p* = 0.002) in the lesions; however, Mφ numbers displayed no obvious difference between the two groups (Figure 6).

### Pathological examinations of Liver

**Figure 7:**
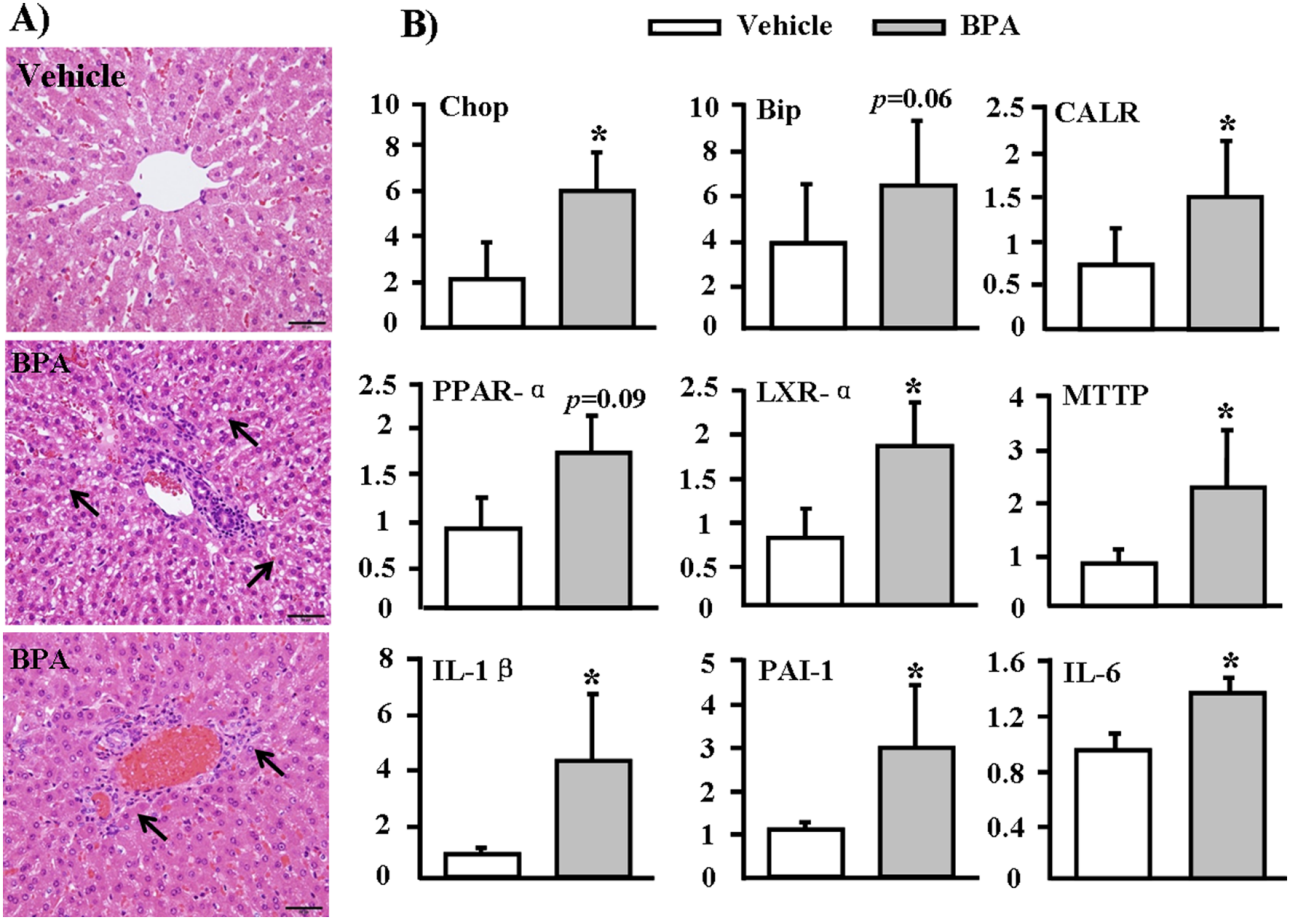
Pathological changes and mRNA expression in the livers of WHHL rabbits at 12 weeks. (A) Representative micrographs stained with H&E showed steatosis (middle) and inflammatory cells infiltration (lower) in the BPA-exposure group. Arrows highlight steatosis and inflammatory cells. (B) RT-PCR analysis of mRNA expressions in the livers of WHHL rabbits after BPA exposure. The mRNA expression levels of genes related to the ER pathway, lipid metabolism, and liver inflammation in WHHL rabbits treated with 400 µg/kg BPA for 12 weeks were analyzed using real-time RT-PCR. Expression levels are expressed relative to the data obtained in the vehicle group. Data are expressed as the means ± SD, n = 6 for each group. *p<0.05 vs. vehicles.

Liver histology results revealed focal hepatic injuries in the BPA-exposed groups, including mild steatosis (Figure 7A, middle) and inflammatory cell infiltration (Figure 7A, bottom). To investigate whether BPA affected hepatic gene expression, we performed real-time RT-PCR analysis, focusing on 3 pathways that may be involved in BPA-induced hepatic toxicity. We found that all ER stress genes (Chop, Bip, and CALR) were up-regulated in the livers of BPA-treated WHHL rabbits compared with those in the vehicle group (Figure 7B, top). Furthermore, 6 genes that mediate lipid metabolism and inflammation (PPAR-α, LXR-α, MTTP, IL-1β, PAI-1 and IL-6) were also up-regulated in livers from the BPA group (Figure 7B, middle and bottom).

### Pathological examinations of Adipose tissue

**Figure 8:**
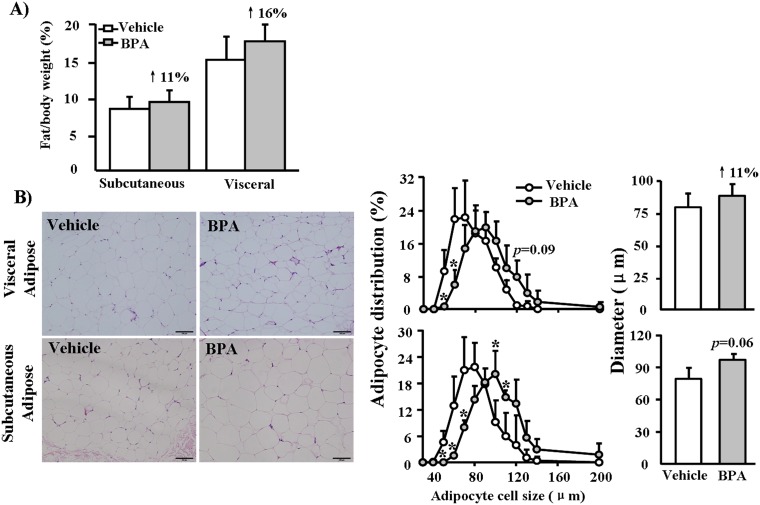
Pathological analysis of adipose in WHHL rabbits at 12 weeks. (A) The total mass of adipose tissue was measured and expressed as the ratio of adipose tissue weight to body weight. (B) Representative micrographs of adipose tissue (H&E-stained specimens), morphometric analysis of adipocyte cell size distribution and mean diameters in the vehicle and BPA-exposure. (400 µg/kg) groups. Data are expressed as the means ± SD, n = 6 for each group. *p<0.05 vs. vehicles.

We found that total weights of subcutaneous and visceral adipose tissues were increased by 11% and 16%, respectively, in the BPA-exposure groups compared to the vehicle group, but these differences were not statistically significant (Figure 8A). Morphometric analysis showed that adipocyte sizes in both visceral and subcutaneous regions of the BPA-exposure groups shifted toward a large-cell population (meaning that large-sized cells predominated) compared to the vehicle group and that the average diameter was also slightly larger in the BPA-exposure groups (Figure 8B).

### Pathological examinations of Heart

**Figure 9:**
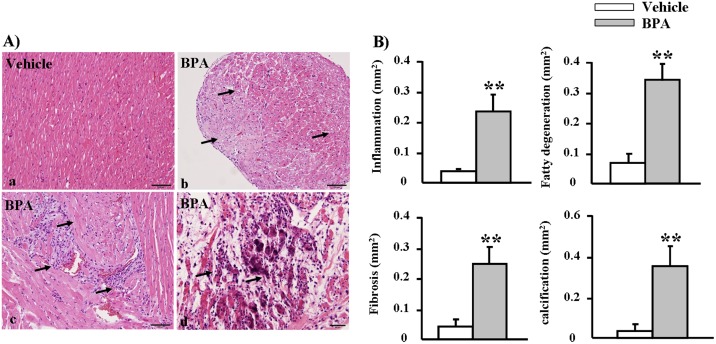
Pathological analyses of WHHL rabbit hearts at 12 weeks. (A) Heart specimens stained with H&E show disarrayed myocardiocytes along with (b) fatty degeneration (c) inflammatory cell infiltration, fibrosis, and (d) calcification in the BPA-exposure group. (B) The lesion areas in the vehicle and BPA-exposure groups were quantified using an image analysis system. Data are expressed as the means ± SD, n = 6 for each group. **p<0.01 vs. vehicles.

In histological and morphometric analyses, the hearts from BPA-exposure groups displayed cardiac damage accompanied by fatty degeneration, mononuclear cell infiltration and fibrosis. In some areas, myocardiocytes showed focal necrosis associated with calcium deposition or calcification (Figure 9). These findings indicate that chronic BPA exposure may directly lead to myocardial damage.

### Cultured cell experiments

**Figure 10:**
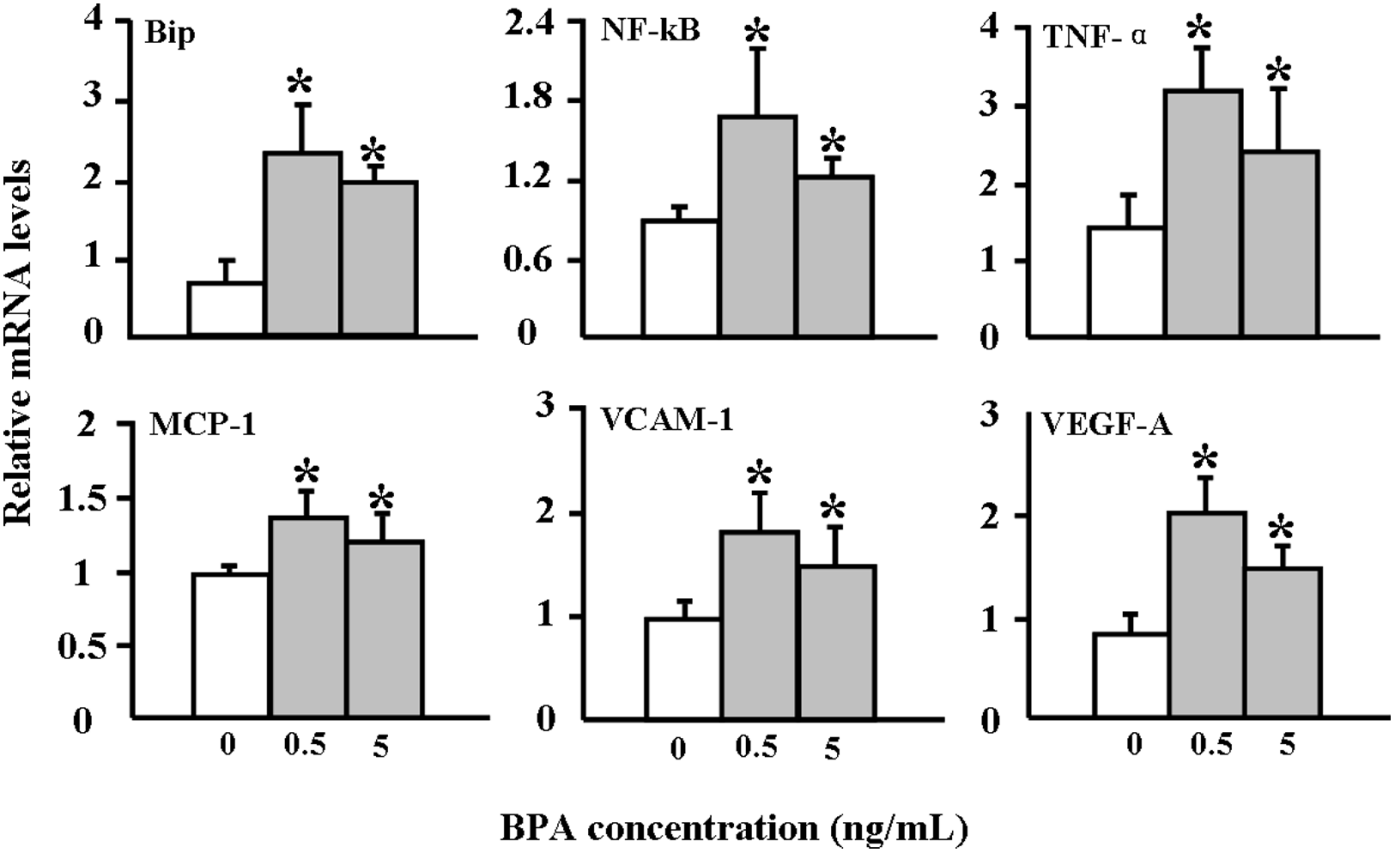
Effects of BPA incubation on ER stress and inflammatory responses in HUVEC cells. Cultured HUVECs were incubated with either vehicle or BPA (0.5 and 5 ng/mL) for 24 hr, after which total RNA was extracted for RT-PCR analysis. The analysis was performed three times, and each assay was performed in triplicate. Representative data are shown as the means ± SD. *p<0.05 vs. vehicles.

**Figure 11:**
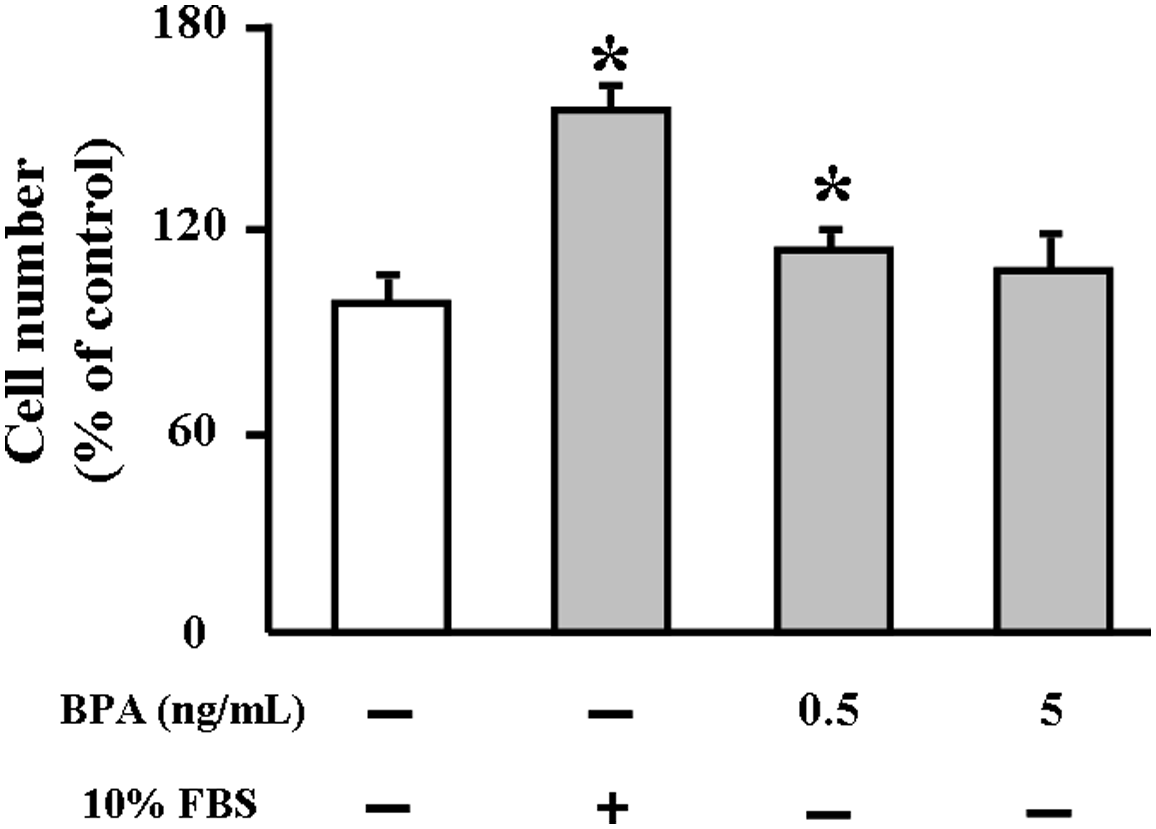
Rabbit aortic smooth muscle cells (SMCs) proliferation. SMCs were treated with either vehicle or BPA (0.5 and 5 ng/mL) for 24 h and 10% fetal bovine serum was used as a positive control. Cell proliferation was determined by CCK-8 assay. Five replicates were performed in each assay. Data are represented as % of control. *p<0.05 vs. vehicles.

The finding that BPA exposure enhances atherosclerosis in WHHL rabbits prompted us to examine the possible mechanisms involved. We investigated the effects of BPA on HUVECs and rabbit aortic SMCs in vitro. BPA exposure did not affect cellular viability of HUVECs under such conditions (data not shown); however, incubation with BPA (0.5 and 5 ng/mL) for 24 hr up-regulated the expression of ER stress (Bip) and inflammatory reactions (MCP-1, NF-κB, TNFα, VCAM-1, and VEGF-A) related genes (Figure 10). Moreover, the SMC number was slightly increased after 0.5 and 5 ng/mL BPA exposure for 24 hr (Figure 11).

## Discussion

The present study demonstrates for the first time that treatment with BPA (400 µg/kg body weight) for 12 weeks led to the enhancement of aortic and coronary atherosclerosis in WHHL rabbits. BPA-treated WHHL rabbits exhibited more advanced lesions and increased numbers of smooth muscle cells. BPA could be rapidly absorbed and eliminated in WHHL rabbits within 24 hr, resulting in serum BPA levels in WHHL rabbits that were not only comparable with the typical range found in humans but were also consistent with findings in rhesus monkeys and CD-1 mice [1], [27], suggesting that the serum levels of BPA could not predict the actual levels and toxic effects of BPA exposure in WHHL rabbits, even with large intakes of BPA from diet [35]. Although we controlled the background BPA contamination strictly and were unable to detect BPA in the laboratory blank samples (below the detection limits), there are still some BPA residuals in serum similar to those of normal humans in the vehicle group, possibly derived from other experimental materials or procedures such as oral gavage and blood drawing. This may also suggest that BPA exposure are unavoidable from various routes in the laboratory environment, which is an elusive laboratory challenge and require to be minimized as much as possible in the future study [36]. However, it is notable that the detectable levels of BPA similar to those of normal healthy humans in the vehicle group showed negligible effects on the WHHL rabbits, which is consistent with that in the general population [37]. On the other hand, the C_max_ of BPA (3.41 ng/mL) reached in the serum of WHHL rabbits at 0.5 hr after BPA exposure, which is close to that in the old people with atherosclerosis (3.76 ng/mL), may be associated with the enhancement of atherosclerosis [9]. Such serum BPA levels did not significantly affect body weights and plasma lipid levels in WHHL rabbits, suggesting that BPA affects the arterial wall directly rather than by mediating plasma lipids as reported in mice [13]. These observations are consistent with reports that treatment of HUVECs with similar amounts of BPA induced up-regulation of ER stress and inflammation, both of which are involved in the pathogenesis of atherosclerosis [38].

Although the mechanisms responsible for increased SMCs in lesions of BPA-treated WHHL rabbits are unknown, we speculate that BPA may modulate the proliferation of SMCs. BPA treatment led to a mild increase of SMCs *in vitro* (Figure 11), suggesting that BPA effect on SMC growth may be indirect, possibly through mediation of growth factors derived from endothelial cells [39]. Previous studies have shown that BPA can influence cellular transport mechanisms by mediating Ca^2+^ channels in some cell types [40], [41]. In cultured human coronary SMCs, BPA has been shown to activate Maxi-K ion channels, which play critical roles in regulating smooth-muscle excitability [42]. Nevertheless, it is not clear whether the BPA-induced SMCs would affect the plaque stability because the lesions are also characterized by having much more lipid-rich and necrotic cores (Figure 5). Meanwhile, BPA exposure affected the functions of endothelial cells, inducing the up-regulation of surface adhesion molecules (VCAM-1), which can increase permeability, enhance leukocyte adhesion, and promote monocyte emigration, contributing to the development of atherosclerosis [43]. Moreover, BPA exposure could also induce various inflammation markers in the endothelial cells and increase the proinflammatory cytokines levels (eg, TNF-α and IL-6) in the serum, all of which may cause the apoptosis of intimal smooth muscle cell and breakdown of matrix [43]. Therefore, it will be necessary to investigate whether BPA can affect the rupture of atherosclerotic plaques.

In addition to BPA’s direct effects on the arterial wall, enhanced atherosclerosis in BPA-treated WHHL rabbits may be attributed to other pathological actions induced by BPA. For example, we found that BPA exposure increased adiposity and induced hypertrophy in adipocytes in both subcutaneous and visceral adipose tissue but that body weight remained unchanged. It has been reported that adipose tissue is the primary target of BPA exposure [44] and that perinatal or postnatal exposure to BPA induces adipocyte hypertrophy in mice and rats that is accompanied by lipogenic gene up-regulation [45], [46]. BPA, a lipophilic compound, can accumulate in adipose tissue, and detectable levels have been found in 50% of breast adipose tissue samples from women [47]. Thus, BPA accumulation in adipose tissue may constitute a secondary source of internal BPA exposure [48].

Moreover, BPA can affect adipocyte differentiation in cultured 3T3-L1 pre-adipocytes, leading to abnormal lipid metabolism [49]. Furthermore, BPA exposure can affect some factors, such as adipokines and cytokines, which are secreted from adipose tissue and can influence the functions of endothelial cells, SMCs, and Mφ in vessel walls [50], [51]. This may constitute another mechanism for BPA-induced enhancement of atherosclerosis [52].

In addition to its effects on adipose tissue, BPA exposure could also impose toxic effects on the liver, including steatosis and inflammatory cell infiltration, but plasma hepatic-marker levels were not increased (data not shown). BPA is rapidly catabolized in the liver within 24 hr, and so the accumulation of high levels of BPA in the liver is certainly detrimental to hepatocyte functions. The pathophysiological significance of BPA-induced amplification of genes related to ER stress, inflammation and lipid metabolism in the liver remains unknown; however, abnormal liver functions accompanied by increased adiposity could certainly contribute significantly to the development of atherosclerosis [50], [53], [54].

An important observation in this study is cardiac damage in BPA-treated WHHL rabbits. Although they require verification, several possible mechanisms may be at play. First, BPA can directly change cardiac functions, as demonstrated by reports that BPA infusion causes ventricular arrhythmias in isolated rat hearts [14], [55]. Second, BPA-induced cardiac damage may be attributed to ischemic effects, as evidenced by BPA-enhanced coronary atherosclerosis in WHHL rabbits. It remains to be seen whether BPA can induce spasms in coronary arteries as a result of BPA-induced up-regulation of ER stress and inflammatory response genes, as observed in cultured endothelial cells in the current study.

## Conclusions

BPA is rapidly catabolized in WHHL rabbits within 24 hr, and serum levels of residual BPA were not significantly increased, suggesting that serum levels of BPA cannot predict the actual levels and toxic effects of BPA exposure [56]. Nevertheless, BPA exposure led to increased atherosclerosis in aortic and coronary atherosclerosis. Although the molecular mechanisms are still not completely understood, increased adiposity and impaired liver and cardiac functions induced by BPA treatment may be involved in the enhancement of atherosclerosis. Taken together, our results suggest that BPA exposure may increase susceptibility to atherosclerosis in the setting of hyperlipidemia.

## Supporting Information

Table S1 **Primer sequences for quantitative real-time PCR in vivo and in vitro studies.**
(PDF)

Technical Description S1 **Technical description of methods.**
(PDF)
