# Supplementary material for: Bisphenol A Exposure Enhances Atherosclerosis in WHHL Rabbits
Source: PLoS One. 2014 Oct 21;9(10):e110977. doi: 10.1371/journal.pone.0110977 (PMC4205098; doi:10.1371/journal.pone.0110977)
Supplement: Table S1 — Primer sequences for quantitative real-time PCR in vivo and in vitro studies. (PDF) [file pone.0110977.s001.pdf]

Table S1. Primer sequences for quantitative real-time PCR in vivo and in vitro studies.

Gene name	5'-Primer	3'-Primer
**Rabbit**		
Bip	ACCTGGTGCTGGATGTATGTC	GAGCAGGGGGAATTCCAGTCAGA
CALR	CGGCGATCAGGAGAAAGATAAA	GCTGTCGTAGGCATAGATGTTAG
Chop	GATGAAAATGGGGGTACCTATGTCTC	GCTGCGCACTGGCCACTCTGTT
GAPDH	ATCACTGCCACCCAGAAGAC	GTGAGTTTCCCGTTCAGCTC
IL-1β	GGAGAGCTCTTTCCCACCAG	TGGGTACCAAGGTTCTTTGAA
IL-6	AGACGACCACGATCCACTTC	CAGGATGGTGTGTTCTGACC
LXR-α	CAGATCCGCCTGAAGAAACT	CTCCCGGTTGTAGCTGAAAT
MTTP	GTCTTCCAGAGCGAGTGTAAAG	GGCAAATCCACAGGCATAAAG
PAI-1	ACGGTCAAGCAGGTGGACT	GAGGGCATTCACCAGCAG
PPAR-α	TACGAGGCCTACCTGAAGAA	CGACGACAGCATCGTGAATA
**Human**		
Bip[1]	CGGGCAAAGATGTCAGGAAAG	TTCTGGACGGGCTTCATAGTAGAC
GAPDH	GGAGAAGGCTGGGGCTCAT	TGATGGCATGGACTGTGGTC
MCP-1	GTGGTCCATGGAATCCTGAA	CAGCCAGATGCAATCAATGC
NF-kB	TGGGAATGGTGAGGTCACTCT	TCCTGAACTCCAGCACTCTCTTC
TNF-α	TGTGTGGCTGCAGGAAGAAC	GCAATTGAAGCACTGGAAAAGG
VCAM-1[2]	ACAGAAGAAGTGGCCCTCCAT	TGGCATCCGTCAGGAAGTG
VEGF-A	CCTGGAGCGTATGTGACAAGCC	TCCTGGTGAGAGATCTGGTTCCC

Bip, binding immunoglobulin protein; *CALR*, calreticulin; *Chop*, C/EBP homologous protein; *GAPDH*, glyceraldehyde 3-phosphate dehydrogenase; *IL-6*, interleukin-6; *LXR-α*, liver X receptor alpha; *MCP-1*, monocyte chemotactic protein 1; *MTTP*, microsomal triglyceride transfer protein; *NF-kB*, nuclear factor-kappa B; *PAI-1*, plasminogen activator inhibitor-1; *PPAR-α*, proliferator-activated receptor alpha; *TNF-α*, tumor necrosis factor alpha; *VCAM-1*, vascular cell adhesion molecule-1; *VEGF-A*, vascular endothelial growth factor A

References:

1. Jian Z, Li J-B, Ma R-Y, Chen L, Wang X-F, et al. (2012) Pivotal role of activating transcription factor 6α in myocardial adaptation to chronic hypoxia. Int J Biochem Cell Biol 44: 972-979.
2. Angel-Morales G, Noratto G, Mertens-Talcott S (2012) Red wine polyphenolics reduce the expression of inflammation markers in human colon-derived CCD-18Co myofibroblast cells: Potential role of microRNA-126. Food Funct 3: 745-752.
