# Supplementary material for: Bisphenol A Exposure Enhances Atherosclerosis in WHHL Rabbits
Source: PLoS One. 2014 Oct 21;9(10):e110977. doi: 10.1371/journal.pone.0110977 (PMC4205098; doi:10.1371/journal.pone.0110977)
Supplement: Technical Description S1 — Technical description of methods. (PDF) [file pone.0110977.s002.pdf]

Technical Description S1

Unconjucted BP A serum levels detection. At beginning, 30 μL 50 ng/mL $^{13}C_{12}$BPA (Cambridge Isotope Laboratories Ins, MA, USA.) was added to each sample of 500 μL serum as an internal standard. Next, 3 mL of ethyl acetate was added to extract BPA twice used by ultrasonic bath for 15 min. Then, the homogenate was vortexed for 30s and centrifugated at 4 ◦C for 10 min at 3000×g to separate the organic and aqueous layers. All of the extracts from the organic layer were combined and evaporated under a gentle stream of nitrogen. Subsequently, in order to improve the sensitive of detection and reduce the background contamination, derivative reaction was performed as follows: 500 μL acetone, 100 μL dansyl chloride (1 mg/mL, dissolved in acetone), and 100 μL Na_2CO_3 buffer solution (0.1M, pH = 10.5) were added to each sample and incubated at 50 ◦C for 30 min in the ultrasonic bath. Afterwards, 1 mL MQ water, which was filtered by SPE column (Oasis HLB 5 cc, 200 mg, Waters, USA) to reduce the background BPA levels less than zero, was added and the homogenate was extracted and centrifugated again as the same procedure mentioned above. All of the extracts from the organic layer were combined and evaporated under a gentle stream of nitrogen before reconstitution in 300 μL methanol, which was centrifugated at 12,000×g for 10 min at 4 ◦C and then 200 μL supernate was transferred into an auto sampler vial for LC-MS/MS analysis. All glassware used in the experiment was washed carefully and baked for 4 h at 500 °C to remove any residual BPA, other interfering chemicals or water.

The mobile phase contained 0.1% formic acid water solution (A) and 100% methanol (B), the flow rate was maintained at 600 μL/min. The gradient was increased from 55 % to 95 % B after 5.06 min and maintained for 5 min. 20 μL sample was injected into a kinetex 2.6 μ C_{18} 100A column (100 mm × 4.6 mm, 2.5 μm particle size, Phenomenex, USA). The temperature of the column was 35 ◦C. The multiple reaction monitoring (MRM) mode was operated in the MS/MS procedure, and the mass transition monitored ions were selected as follows: 707→171 for $^{13}C_{12}$BPA, 695→171.2 for BPA. Quantification of BPA in samples was performed using a 8 points external calibration curve with BPA contrations ranged from 0 to 100 ng/mL. The standard curves of BPA were linear and the correlation coefficient was 0.9898. The extraction recovery percentage of BPA was measured using 3 blank of 500 μL

serum spiked with 3 known amounts of BPA (0.5, 5 and 50 μg/L) and 5 μg/L $^{13}C_{12}$BPA as an internal standard. Each sample contained 3 independent replicates and extracted using the same procedure as samples. The concentrations of BPA were calculated from standard curves, and the mean extraction recovery of BPA through the entire analytical procedure ranged from 81% to 113%. The detection limit of the method for BPA was 0.02~0.27 ng/mL. We used methanol (HPLC) as blank samples and were prepared in triplicate, which were extracted and pretreated following the same procedure as serum samples. The background BPA levels were not able to be detected (below the detection limits) in the blank samples through the whole experimental process.

Analysis of serum cytokines levels. The serum cytokines levels of TNFα and IL6 were analyzed using rabbit TNFα and IL6 ELISA kits (Cloud-Clone Corp.& USCN, Houston, USA) according to the manufacturer's instructions. In brief, 100 μL standards and serum samples were added to the microtiter plate wells with a biotin-conjugated antibody specific to TNFα or IL6. After incubated for 2 hr at 37℃, the liquid of each well was removed. Then 100 μL of prepared detection reagent A was added and incubated for 1 hr at 37℃ after covering it with the plate sealer. Afterwards, all the solution was removed and each plate well was washed 3 times using the 350 μL prepared 1×wash buffer. The remaining wash buffer should be removed completely by blotting it against absorbent paper. Then 100 μL of prepared detection reagent B was added to each well and incubated for 1 hr at 37℃. The remove and wash process was repeated for total 5 times as described above. Subsequently, 90 μL of substrate solution was added to each well and incubated for 20 min at 37℃ in the dark environment. When the obvious blue color was appeared in the first three standard samples, 50 μL of stop solution was added to each well and the liquid changed from blue to yellow at once. Then, run the microplate reader and the measurement was conducted at 450 nm immediately. The standard curve was constructed by plotting the mena O.D. and concentration for each standard and draw a best fit curve through the points. The concentration of TNFα and IL6 in the serum was then determined by comparing the O.D. of the samples to the standard curve.

HUVEC culture and treatments. HUVEC were grown in RPMI 1640 medium supplemented with 10% (V/V) fetal bovine serum (FBS) (Gibco). All cell cultures were

maintained in a humidified atmosphere containing 5% CO_2 (V/V) at 37 ℃. For experiments, cells were seeded in six-well plates. In the light of the rapid elimination of BPA levels in the serum of WHHL rabbits, the exposure time was lasted for 24 hr. After that, the old medium was changed by the fresh medium containing serial dilutions of BPA (0, 0.5, 5 ng/mL). BPA and thapsigargin were both dissolved in dimethyl sulfoxide (DMSO) and the final concentration of DMSO in medium was controled at 0.1%, v/v.

MTT viability assay. The cell viability of HUVEC after BPA exposure was assessed by MTT assay. Briefly, the cells were seeded in 96-well plates and exposed by BPA for 6 hr. At the end of the expsoure, 20 μL MTT (0.25 mg/ml) was added to each well and incubated for another 4 h. Then the medium was removed and 150 μL DMSO was added. After that, the plates were shaked slowly for 10 min to ensure the purple formazan precipitate was dissolved with DMSO, and the color intensity was measured at 550 nm with a SpectraMAX M5 microplate reader (Molecular Devices, Sunnyvale,CA, USA).

Cell total RNA preparation and real-time PCR. Total RNA was extracted using the EZNA total RNA kit Ⅱ (Omega, Doraville, GA, USA). Equal amount of RNA was then reverse transcribed using PrimeScript TM RT-PCR Kit (Takara, Dalian, China) according to the manual. Real-time PCR was carried out on a LightCycler 480 Instrument (Roche, Rotkreuz, Switzerland) using SYBR Premix Ex Taq kit (TaKaRa, Dalian, China PR) following the manufacturer’s instructions. All quantifications were conducted with 36B4 as an internal standard, and the relative amount of mRNA was calculated using the $2^{-\Delta\Delta CT}$ method.

Smooth muscle cell culture. The aortas of Japanese white rabbits were isolated and cleaned under sterile condition. Series of about 1 mm^2 pieces of aortic medial parts were sectioned and were incubated with PBS supplemented with collagenase Ⅰ (1 mg/mL) and elastase (0.25 mg/mL, Worthington Biochemical Corp., Lakewood, NJ) for 60 minutes at 37 ℃. Then the supensions of smooth muscle cell (SMC) were centrifuged at 1,500 rpm for 5 minutes and the cell pellet was resuspended in 5 mL of DMEM-F12 (Nacalai Tesque, Inc., Kyoto) contained 10 % fetal bovine serum, 100 U/mL penicillin and 100 μg/mL streptomycin (Gibco/Invitrogen, Carlsbad, CA). The SMCs used for experiments within 5th passages.

Cell Proliferation Assay. The cell proliferation assay of the SMCs caused by BPA treatment was used by cell counting kit-8 (Dojindo Molecular Technologies, Inc., Rockville, USA) following the manufacturer's instructions. In brief, cells were seeded (6×10^4 cell/mL) in 96 well plates (100 μL/well) for 24 hr. Then the cells were starved for 24 hr without fetal bovine serum. Afterwards, the old medium was replaced by the fresh medium supplemented with serial dilutions of BPA (0, 0.5, 5 ng/mL) and the 10 % fetal bovine serum was taken as a positive control. After incubating for 24 hr, 10 μl of the CCK-8 solution was added to each well of the plate. After incubated in the incubator for 2 hours, the absorbance was measured at 450 nm using a microplate reader.
